# “Dynamic Range” of Inferred Phenotypic HIV Drug Resistance Values in Clinical Practice

**DOI:** 10.1371/journal.pone.0017402

**Published:** 2011-02-24

**Authors:** Luke C. Swenson, Graham Pollock, Brian Wynhoven, Theresa Mo, Winnie Dong, Robert S. Hogg, Julio S. G. Montaner, P. Richard Harrigan

**Affiliations:** 1 BC Centre for Excellence in HIV/AIDS, St. Paul's Hospital, Vancouver, British Columbia, Canada; 2 Division of AIDS, Faculty of Medicine, University of British Columbia, Vancouver, British Columbia, Canada; 3 Faculty of Health Sciences, Simon Fraser University, Burnaby, British Columbia, Canada; University of Rochester, United States of America

## Abstract

**Background:**

‘Virtual’ or inferred phenotypes (vPhenotypes) are commonly used to assess resistance to antiretroviral agents in patients failing therapy. In this study, we provide a clinical context for understanding vPhenotype values.

**Methods:**

All HIV-infected persons enrolled in the British Columbia Drug Treatment Program with a baseline plasma viral load (pVL) and follow-up genotypic resistance and pVL results were included up to October 29, 2008 (N = 5,277). Change from baseline pVL was determined as a function of Virco vPhenotype, and the “dynamic range” (defined here by the 10th and 90th percentiles for fold-change in IC_50_ amongst all patients) was estimated from the distribution of vPhenotye fold-changes across the cohort.

**Results:**

The distribution of vPhenotypes from a large cohort of HIV patients who have failed therapy are presented for all available antiretroviral agents. A maximum change in IC_50_ of at least 13-fold was observed for all drugs. The dideoxy drugs, tenofovir and most PIs exhibited small “dynamic ranges” with values of <4-fold change observed in >99% of samples. In contrast, zidovudine, lamivudine, emtricitabine and the non-nucleoside reverse transcriptase inihibitors (excluding etravirine) had large dynamic ranges.

**Conclusion:**

We describe the populational distribution of vPhenotypes such that vPhenotype results can be interpreted relative to other patients in a drug-specific manner.

## Introduction

HIV drug resistance testing assists clinical decision-making in the selection of antiretroviral therapy [1], [2], and is routinely used as a guide to future treatment options for HIV-infected patients who develop virological treatment failure [3], [4]. A number of genotypic resistance interpretation systems are available based on either rules-based algorithms or “virtual phenotypes.” However, the lack of concordance (up to ∼20%, [5]) among the data generated using these systems clearly signals a need for standardization and a context from which to approach an individual patient's result [6].

Currently, genotypic rather than phenotypic tests are commonly used for drug resistance testing, largely due to the former's lower cost and faster turn-around time. However, inferring phenotypes from genotypic testing remains a challenge due to subtle changes in viral replication and patterns of mutational profiles. Consequently, the various genotype-phenotype interpretation algorithms have met with variable success [1], [7], [8], [9].

### Cut-offs

The interpretation of either a virtual or real phenotype is based primarily on a “cut-off” value, which defines a threshold between a susceptible wild-type phenotype and a reduced drug susceptibility (ie, resistant) phenotype. Initially, cut-offs were based on the reproducibility of the assay (“technical cutoffs”). With this system, all antiretrovirals were assigned the same (relatively arbitrary) cut-off value, and if the fold-change in drug concentration required to inhibit the virus was greater than this value, the variants were considered to have reduced susceptibility compared to wild type.

This was subsequently refined by the use of epidemiologically derived cut-offs from the distribution of wild-type susceptibilities in large numbers of antiretroviral-naïve patients [10]. These biological cut-offs take advantage of the natural variation in drug susceptibility present in non-drug-exposed HIV variants [10] and are more clinically relevant than arbitrary values for assay reproducibility. However, biological cut-offs are not derived from data of clinical responses to antiretroviral agents and may therefore lack clinical relevance.

In a phenotypic resistance assay, the degree of resistance is defined using the median inhibitory concentration (IC_50_). IC_50_ is the concentration of a drug required for a 50% inhibition of viral replication *in vitro* [11]. vPhenotypes are assembled using large databases of phenotypic resistance data matched to genotypic viral sequences derived from patients. The resistance level of a vPhenotype is based on observed phenotypic fold-changes (FC) in IC_50_ associated with the replication of a virus with a given sequence. Higher fold-changes represent lower susceptibility to the drug relative to the reference strain. In other words, if a virus showed a two-fold increase in IC_50_ (essentially a vPhenotype value of 2), this would indicate that twice as much drug would be required to inhibit viral replication by one-half, compared to a wild-type virus. Thus, the phenotypic resistance level of a patient's virus can be predicted from the vPhenotype associated with the viral sequence.

However, cut-offs based on the actual *in vivo* virological response to a regimen may better inform the interpretation of resistance data [12] and may provide a more accurate clinical prognosis for patients on long-term antiretroviral therapy. Clinical cut-offs (CCO) can be established by using vPhenotypes to determine clinically relevant phenotypic fold-change resistance levels [13]. In this approach, the cutoffs are defined not by an *in vitro* indicator, but by actual virologic responses to therapy in patients with drug resistant HIV. The lower CCO indicates the point at which virologic response to an agent begins to be compromised, and the upper CCO indicates the point where response to the agent is nearly completely abolished [13].

In addition to knowing whether a patient's inferred phenotype is above or below a given clinical or biological cut-off, we feel that it may be useful to place the results against the spectrum of other patients experiencing virological treatment failure. By comparing vPhenotype levels across such a dataset, frequency distributions of the various resistance levels may be constructed, revealing a profile of the resistance generally experienced by patients during drug treatment. This serves as a good point of comparison for assessing the severity of a patient's vPhenotypic resistance level in the context of other patients undergoing treatment. By combining all patient-derived resistance data together, the drug's overall “dynamic range” can be determined as the range of vPhenotype scores between which a majority of patient samples fall.

Here we present data on the distribution of vPhenotypes from a large cohort of patients failing therapy in British Columbia, Canada. We have determined minimum and maximum virtual phenotypic susceptibility, as well as the “dynamic range” of susceptibility for all licensed antiretroviral agents.

## Methods

### Study population

We evaluated all HIV-infected adults who enrolled in the British Columbia (BC) Drug Treatment Program between 1996 and 2008 with a baseline plasma viral load (pVL) >1000 HIV RNA copies/mL until 2001 and >250 copies/mL thereafter; and for whom at least one follow-up sample with genotypic drug resistance data was available. Samples were evaluated from a total of 5,277 patients, most of whom had multiple samples (median 2; inter-quartile range: 1–5). From these samples, a total of 19,611 vPhenotype results were obtained by genotyping of the HIV protease (PR) and reverse transcriptase (RT) regions for HIV drug resistance mutations at the BC Centre for Excellence in HIV/AIDS.

### HIV RNA extraction and drug resistance analysis

Drug resistance testing was performed on physician-requested samples with pVL as defined above. HIV RNA was extracted from frozen plasma samples using guanidinium-based lysis buffer followed by isopropanol/ethanol washes or by automated extraction using a NucliSENS easyMAG (bioMerieux). Amplification of the PR and RT regions was performed using nested RT-PCR and sequenced in both the 5′ and 3′ directions on an ABI 3100 or 3730 automated sequencer. Sequence data were analyzed using Sequencher (Genecodes) or RE_Call (BC Centre for Excellence in HIV/AIDS [14], [15], [16]) sequencing software. Nucleotide mixtures were identified if the secondary peak height exceeded 20% of the dominant peak height. Sequences were aligned to HIV-1 subtype B reference strain HXB2 (Genbank Acc. No. K03455) using a modified NAP algorithm [17]. Results of the genotyping analysis were reported as amino acid changes in the HIV protease and reverse transcriptase genes relative to HXB2.

### Generation and analysis of vPhenotypes

Virco (Mechelen, Belgium) converted the genotypic sequence data to Virtual Phenotypes. The virco®TYPE versions used in this analysis varied over time, where the specific version used depended on the version in place at the time of testing. vPhenotype results were expressed as percentile ranks of fold-change in IC_50_ for each antiretroviral drug. Analysis of the distribution of vPhenotypes was performed on two groups: all samples sequenced within the BC Drug Treatment program (N = 19,611), and a subset of about half of these samples (N = 9,606) which had one or more International AIDS Society (IAS) key drug resistance mutation(s) [18]. The dynamic range of vPhenotype scores for each agent was defined as the range between 10^th^ and 90^th^ percentiles of values. These percentiles were somewhat arbitrarily chosen, but are illustrative in that they reflect the range between which a majority of patient samples falls, while excluding outlier values. We have also presented the 1^st^ & 99^th^, and 5^th^ & 95^th^ percentiles for the reader's convenience.

### Ethics Statement

Ethical approval was granted by the Providence Health Care/University of British Columbia Ethics Board. All data were analysed anonymously. Requirement for consent was waived by the Ethics Board because the analysis involved no more than minimal risk to subjects.

## Results

### Distribution of virtual phenotypes

**Figure 1:**
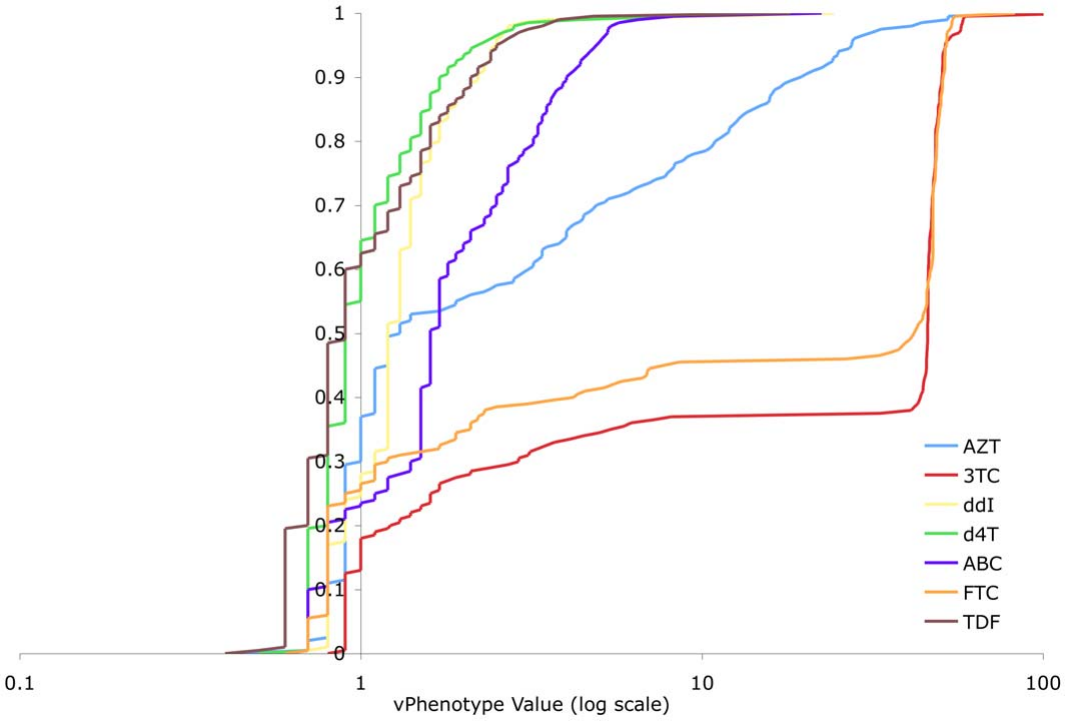
Distribution of Resistance to NRTIs (BC vPhenotypes with 1 or More IAS Key Mutation). The distribution of the vPhenotype value (log transformed) for nucleoside/nucleotide reverse transcriptase inhibitors across samples tested in British Columbia where at least 1 International AIDS Society key mutation was present. Percentiles indicated include every half percentile, as the minimum and maximum values for each agent. AZT  =  zidovudine, 3TC  =  lamivudine, ddI  =  didanosine, d4T  =  stavudine, ABC  =  abacavir, FTC  =  emtricitabine, TDF  =  tenofovir. The individual distributions may be grouped into 3 general categories: 3TC/FTC, AZT, and other NRTIs.

**Figure 2:**
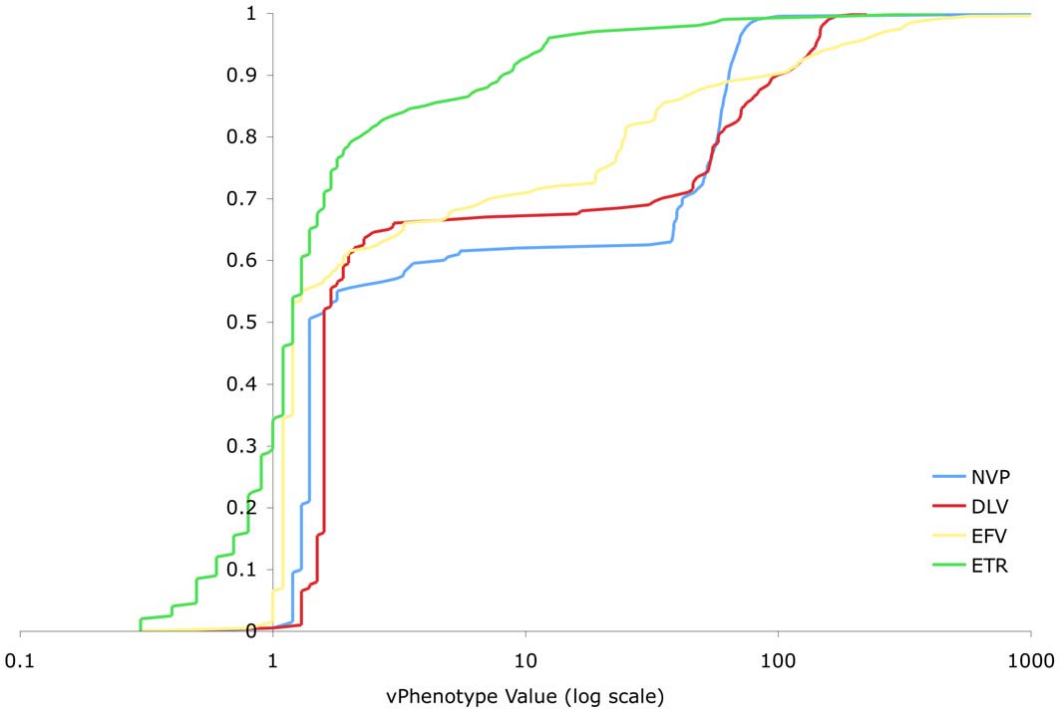
Distribution of Resistance to NNRTIs (BC vPhenotypes with 1 or More IAS Key Mutation). The distribution of the vPhenotype value (log transformed) for non-nucleoside reverse transcriptase inhibitors across samples tested in British Columbia where at least 1 International AIDS Society key mutation was present. Percentiles indicated include every half percentile, as the minimum and maximum values for each agent. Note that the scale of the horizontal axis extends to 1000 rather than 100 for Figures 1a and 1c, reflecting the higher maximum fold-change values observed for the NNRTI drug class. NVP  =  nevirapine, DLV  =  delavirdine, EFV  =  efavirenz, ETR  =  etravirine.

**Figure 3:**
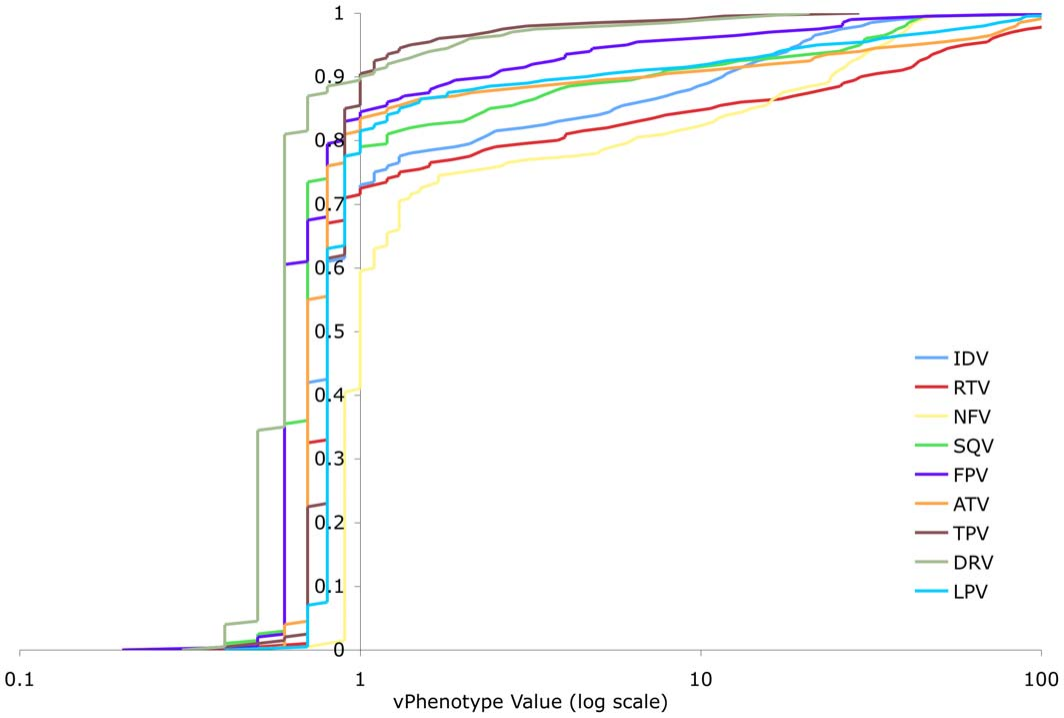
Distribution of Resistance to PIs (BC vPhenotypes with 1 or More IAS Key Mutation). The distribution of the vPhenotype value (log transformed) for protease inhibitors across samples tested in British Columbia where at least 1 International AIDS Society key mutation was present. Percentiles indicated include every half percentile, as the minimum and maximum values for each agent. IDV  =  indinavir, RTV  =  ritonavir, NFV  =  nelfinavir, SQV  =  saquinavir, FPV  =  fosamprenavir, ATV  =  atazanavir, TPR  =  tipranavir, DRV  =  darunavir, LPV  =  lopinavir.

Virtual phenotypes were collected for a total of 19,611 samples from 5,277 different patients. Results were analyzed in two sets: (I) all samples submitted for virtual phenotyping; and (II) a subset of group I for which at least one key IAS resistance mutation [18] was identified. The distribution of vPhenotypes for all samples tested in BC is summarized in Table 1. The distribution of vPhenotypes for samples with one or more IAS key mutation (N = 9,606) is shown for each drug class in Figures 1, 2, and 3, and summarized in Table 2. Overall, the shapes of the distributions from both groups resemble each other closely within each drug class. Data are generally presented for the IAS key mutation subset for simplicity, to limit the effect of testing of antiretroviral-naïve individuals, as well as to emphasize a more clinically relevant group of patients where at least some degree of drug resistance is certain.

**Table 1:** HIV susceptibility to antiretroviral agents (All BC vPhenotypes).

			Probability distribution of Virtual phenotype (fold-change in IC_50_)(N = 19,611)								
Drug	Lower CCO – Upper CCO	N	Percentile								
			Min	1st	5th	*10th*	50^th^	*90^th^*	95^th^	99th	Max
**AZT**	1.5–11.4	18392	0.4	0.7	0.8	*0.9*	1.1	*11.3*	19.6	41.2	105
**3TC**	1.2–4.6	18432	0.7	0.8	0.9	*0.9*	1.0	*48.4*	50.6	57.5	133.8
**ddI**	0.9–2.6	18177	0.6	0.8	0.8	*0.8*	0.9	*1.7*	2.2	2.7	24.4
**d4T**	1.0–2.3	18281	0.5	0.7	0.7	*0.7*	0.9	*1.4*	1.7	2.8	13.3
**ABC**	0.9–3.5	18135	0.5	0.7	0.7	*0.7*	0.8	*3.2*	4.0	5.4	22.4
**FTC**	3.1*	7915	0.5	0.6	0.7	*0.7*	0.8	*47.5*	50.2	52.6	82.6
**TDF**	1.0–2.3	13799	0.4	0.6	0.6	*0.7*	0.8	*1.5*	2.1	3.3	18.2
**NVP**	6.0*	18538	0.5	1.0	1.2	*1.2*	1.4	*58.0*	64.1	76.1	2152.5
**DLV**	N/A	16624	0.3	1.3	1.3	*1.3*	1.6	*60.9*	109.6	151.4	224.5
**EFV**	3.3*	18491	0.3	0.9	1.0	*1.0*	1.1	*24.9*	105.8	316.3	43341.0
**ETR**	1.6–27.6	1545	0.3	0.4	0.7	*0.8*	1.2	*2*	4.2	22.8	460.0
**IDV/r**	2.3–27.2	18335	0.2	0.6	0.7	*0.7*	0.7	*2.2*	11.6	29.7	145.7
**RTV**	N/A	16498	0.3	0.7	0.7	*0.7*	0.8	*4.1*	32.8	111.5	258.3
**NFV**	1.2–9.4	18325	0.4	0.8	0.9	*0.9*	0.9	*6.5*	24.2	41.2	115.6
**SQV/r**	3.1–22.6	18355	0.3	0.5	0.6	*0.6*	0.7	*1.2*	6.9	39.9	183.9
**FPV/r**	1.5–19.5	6631	0.2	0.5	0.6	*0.6*	0.6	*0.8*	1.2	17.2	121.5
**LPV/r**	6.1–51.2	16293	0.4	0.7	0.7	*0.8*	0.8	*1.0*	3.4	62.0	298.4
**ATV/r**	2.5–32.4	8559	0.5	0.6	0.6	*0.7*	0.7	*0.9*	1.5	76.9	303.8
**TPV/r**	1.5–7.0	6566	0.3	0.6	0.7	*0.7*	0.8	*0.9*	1.0	2.4	29.2
**DRV/r**	10.0–106.9	1853	0.3	0.4	0.5	*0.5*	0.6	*0.7*	0.8	2.7	20.9

Dynamic range indicated by italics.

*A total of 3 drugs have only one biological cut-off (for in vitro susceptibility): FTC, NVP, and EFV.

AZT – zidovudine, 3TC – lamivudine; ddI – didanosine; d4T – stavudine; ABC – abacavir; FTC – emtricitabine; TDF – tenofovir; NVP – nevirapine; DLV – delavirdine; EFV – efavirenz; ETR – etravirine; /r – ritonavir boosted; IDV – indinavir; RTV – ritonavir; NFV – nelfinavir; SQV – saquinavir; FPV – fosamprenavir; LPV – lopinavir; ATV – atazanavir; TPV – tipranavir; DRV – darunavir.

**Table 2:** HIV susceptibility to antiretroviral agents (All BC vPhenotypes with one or more IAS Key mutation).

			Probability distribution of Virtual phenotype (fold-change in IC_50_)(N = 9,606)								
Drug	Lower CCO – Upper CCO	N	Percentile								
			Min	1st	5th	*10th*	50^th^	*90^th^*	95^th^	99th	Max
**AZT**	1.5–11.4	9360	0.4	0.7	0.8	*0.8*	1.3	*19.5*	27.5	52.7	105
**3TC**	1.2–4.6	9402	0.8	0.9	0.9	*0.9*	45.8	*50.5*	51.7	58.2	133.8
**ddI**	0.9–2.6	9149	0.6	0.8	0.8	*0.8*	1.2	*2.2*	2.5	3.5	24.4
**d4T**	1.0–2.3	9251	0.5	0.7	0.7	*0.7*	0.9	*1.7*	2.2	4.4	13.3
**ABC**	0.9–3.5	9105	0.6	0.7	0.7	*0.7*	1.6	*4.0*	4.9	6.5	22.4
**FTC**	3.1*	2829	0.6	0.7	0.7	*0.8*	41.8	*50.9*	52.1	54.1	82.6
**TDF**	1.0–2.3	6435	0.4	0.6	0.6	*0.6*	0.9	*2.1*	2.5	3.8	18.2
**NVP**	6.0*	9513	0.5	1.1	1.2	*1.3*	1.4	*64.0*	69.6	83.0	2152.5
**DLV**	N/A	8866	0.3	1.3	1.3	*1.5*	1.6	*100.0*	141.3	158.3	224.5
**EFV**	3.3*	9468	0.3	0.9	1.0	*1.1*	1.2	*94.8*	181.1	411.0	43341.0
**ETR**	1.6–27.6	500	0.3	0.3	0.5	*0.6*	1.2	*8.2*	11.9	60.8	460.0
**IDV/r**	2.3–27.2	9303	0.2	0.7	0.7	*0.7*	0.8	*11.6*	19.5	37.8	145.7
**RTV**	N/A	8733	0.3	0.7	0.7	*0.7*	0.8	*29.5*	59.3	160.9	258.3
**NFV**	1.2–9.4	9293	0.4	0.8	0.9	*0.9*	1.0	*24.1*	34.2	44.4	115.6
**SQV/r**	3.1–22.6	9323	0.3	0.4	0.6	*0.6*	0.7	*6.9*	29.4	42.8	183.9
**FPV/r**	1.5–19.5	2290	0.2	0.5	0.6	*0.6*	0.6	*2.4*	6.1	27.6	121.5
**LPV/r**	6.1–51.2	7707	0.4	0.7	0.7	*0.8*	0.8	*4.6*	22.1	86.2	298.4
**ATV/r**	2.5–32.4	3071	0.5	0.6	0.7	*0.7*	0.7	*7.1*	42.1	97.3	303.8
**TPV/r**	1.5–7.0	2232	0.3	0.5	0.7	*0.7*	0.8	*1.0*	1.4	9.0	29.2
**DRV/r**	10.0–106.9	586	0.3	0.4	0.5	*0.5*	0.6	*1.0*	1.9	10.5	20.9

Dynamic range indicated by italics.

*A total of 3 drugs have only one biological cut-off (for in vitro susceptibility): FTC, NVP, and EFV.

AZT – zidovudine, 3TC – lamivudine; ddI – didanosine; d4T – stavudine; ABC – abacavir; FTC – emtricitabine; TDF – tenofovir; NVP – nevirapine; DLV – delavirdine; EFV – efavirenz; ETR – etravirine; /r – ritonavir boosted; IDV – indinavir; RTV – ritonavir; NFV – nelfinavir; SQV – saquinavir; FPV – fosamprenavir; LPV – lopinavir; ATV – atazanavir; TPV – tipranavir; DRV – darunavir.

Also shown for each drug is the proportion of patients (with 1 or more IAS resistance mutation) that fall below, between, and above the clinical cut-offs of the Virco vPhenotype reports (Table 3). In general, a majority of these patients have virus that falls below the lower clinical cut off (CCO) for most drugs that have established CCOs: 15/19 drugs (79%). The exceptions are the nucleoside reverse transcriptase inhibitors lamivudine (3TC), emtricitabine (FTC), didanosine (ddI), and abacavir (ABC), with the former two having a majority of patients with vPhenotypes above the upper CCO, and the latter two with a majority of patients falling between the two CCOs. When all 19,611 vPhenotypes were examined, a majority of samples had vPhenotypes below the lower CCO for every agent except ddI (data not shown).

**Table 3:** Proportion of patients falling below, between, and above the Virco Clinical Cut-offs (All BC Virtual Phenotypes with one or more IAS Key mutation).

Drug	Lower CCO – Upper CCO	Proportion <Lower CCO (%)	ProportionBetween CCOs (%)	Proportion>Upper CCO (%)
**AZT**	1.5–11.4	53	28	19
**3TC**	1.2–4.6	19	15	66
**ddI**	0.9–2.6	17	79	4
**d4T**	1.0–2.3	55	41	5
**ABC**	0.9–3.5	21	63	16
**FTC**	3.1*	39	-	61
**TDF**	1.0–2.3	60	31	8
				
**NVP**	6.0*	62	-	38
**EFV**	3.3*	65	-	35
**ETR**	1.6–27.6	68	29	3
				
**IDV/r**	2.3–27.2	80	17	2
**NFV**	1.2–9.4	63	19	18
**SQV/r**	3.1–22.6	86	8	6
**FPV/r**	1.5–19.5	87	9	4
**LPV/r**	6.1–51.2	91	6	3
**ATV/r**	2.5–32.4	88	7	5
**TPV/r**	1.5–7.0	95	4	1
**DRV/r**	10.0–106.9	99	1	0

The drugs DLV and RTV were not included because the Virco clinical cut-offs are not reported for these agents.

*A total of 3 drugs have only one biological cut-off (for in vitro susceptibility): FTC, NVP, and EFV.

The category (below, between, above) where a majority of samples fall is bolded for each agent.

AZT – zidovudine, 3TC – lamivudine; ddI – didanosine; d4T – stavudine; ABC – abacavir; FTC – emtricitabine; TDF – tenofovir; NVP – nevirapine; EFV – efavirenz; ETR – etravirine; /r – ritonavir boosted; IDV – indinavir; NFV – nelfinavir; SQV – saquinavir; FPV – fosamprenavir; LPV – lopinavir; ATV – atazanavir; TPV – tipranavir; DRV – darunavir.

It is worth noting that HIV from patients exhibited a range of “wild-type” phenotypic susceptibility to different drugs that was above or below that seen for the reference laboratory wild-type strain. In other words, the baseline drug susceptibility of “wild-type” viruses varied among different patient-derived HIV strains, and this range of baseline susceptibility should be kept in mind when considering the susceptibility of resistant viruses. For example, the 50^th^ percentile for vPhenotypes of most drugs hovered around 1-fold change (0.6 to 1.6), but with two notable exceptions of >40-fold change for 3TC and FTC (Table 2).

Fold-change in IC_50_ was >1 in a majority of non-nucleoside reverse transcriptase inhibitor (NNRTI) samples and <1 in more than 50% of protease inhibitor (PI) and nucleoside reverse transcriptase inhibitor (NRTI) samples (Table 1 and 2). The range and pattern of vPhenotype distribution varied widely among the different antiretrovirals. Stavudine (d4T), didanosine (ddI), and tenofovir (TDF) displayed some of the lowest maximum changes in vPhenotype susceptibilities (approximately 13- to 25-fold, Table 1) and the narrowest “dynamic ranges,” all falling between 0.7- and 1.8-fold (Table 1). For these drugs there was a modest <2-fold decrease in susceptibility in 90% of samples and <4-fold decrease in susceptibility in 99% of all samples tested. In contrast, the NNRTIs delavirdine (DLV) and nevirapine (NVP) exhibited the broadest dynamic ranges of approximately 1- to 60-fold. The maximum fold-change in IC_50_ for any drug was not representative of the drug's dynamic range. For the drugs studied, the median difference was ∼12-fold between the maximum decrease in susceptibility and the upper limit of the dynamic range, with this difference being most dramatic for the NNRTIs (Table 1).

#### NRTIs

Overall, there are three general distributions of resistance within the NRTI class: 3TC/FTC comprise one group; zidovudine (AZT) comprises another; and the remaining NRTIs (ddI, d4T, TDF, and ABC) comprise a third group (Figure 1). 3TC and FTC both displayed the broadest dynamic ranges, reaching approximately 50-fold decreased susceptibility (more than 10 times greater than the upper limit of dynamic range observed for the other NRTIs, excepting AZT). AZT exhibited an intermediate dynamic range, while the other NRTIs showed a relatively narrow range of resistance.

#### NNRTIs

Two distinct groupings of NNRTI drugs were observed based on the distribution of virtual phenotypes: etravirine (ETR), the newest member of this drug class, constituted one group, while all other NNRTIs (efavirenz [EFV], delavirdine [DLV] and nevirapine [NVP]) comprised the second. ETR exhibited a dynamic range roughly similar to that of the third group of nucleoside agents discussed above (i.e., ddI, d4T, ABC, TDF), while the other NNRTIs displayed dynamic ranges approximately 10 times wider (Table 2, Figure 2). In general, the NNRTIs (excluding ETR) had the widest dynamic ranges of susceptibility and the largest maximum fold-changes in IC_50_ (over 40,000-fold for EFV) of all the drugs studied.

#### PIs

The protease inhibitors all exhibited roughly the same dynamic ranges and distributions of resistance (Tables 1 and 2, Figure 3). Most fell within a relatively narrow dynamic range of <1-fold to approximately 7-fold, though ritonavir (RTV) and nelfinavir (NFV) exhibited larger dynamic ranges extending to >20-fold. Indinavir (IDV) displayed an intermediate dynamic range with an upper limit of 11.6-fold (Table 2). The dynamic ranges of tipranavir (TPV) and darunavir (DRV) were especially low (0.7 to 1.0; and 0.5 to 1.0, respectively), indicating that almost 90% of patient virus in BC was hypersusceptible to these agents – though fewer patients in our program have been treated with them (Tables 1 and 2). For all PIs, the median fold-change in susceptibility was ≤1, indicating a majority of samples having lower susceptibility to these agents compared to the reference strain.

### Comparing the dynamic range of vPhenotypes to clinical cutoffs

The “dynamic range” for most NRTIs was generally comparable to the range between their individual CCOs, with the exception of zidovudine and lamivudine, where the 90^th^ percentile was well above the upper Virco CCO. Conversely, the dynamic ranges of most PIs (excluding nelfinavir) were well within the range of their CCOs, perhaps reflecting the higher genetic barrier to resistance for this drug class. Neither of the two most common NNRTIs nevirapine nor efavirenz has a defined CCO range, so the NNRTIs are excluded from this comparison.

## Discussion

Here we present the distribution of vPhenotypes from a large cohort of HIV-infected individuals who initiated antiretroviral therapy in British Columbia between 1996 and 2008 and who subsequently failed antiretroviral therapy during follow-up. These data clearly indicate that the range of IC_50_ values varies widely among antiretroviral agents regardless of drug class. For example, the upper limit of the dynamic range (90^th^ percentile) varied from 1-fold change for the PIs TPV and DRV to around 100 for the NNRTIs DLV and EFV. The maximum fold-change in IC_50_ varied even more dramatically, ranging from approximately 13 for d4T to more than 43,000 for EFV. Some sequences exceeded the upper limits of the assay's ability to measure vPhenotype. The wide variability in IC_50_ fold-change also points to the value of interpreting variations in susceptibility within the context of a large dataset of other patient samples, such as that described here. We also present the proportion of patients with HIV that falls between and outside the Virco clinical cut-offs for vPhenotype resistance, which provides a good basis for the overall extent of drug resistance to various antiretroviral agents in a typical Western clinical setting.

A raw FC-IC_50_ vPhenotype score is difficult to interpret and is unlikely to be useful clinically, as its meaning may vary depending on the drug under consideration. Interpreting the vPhenotype of a patient-derived virus in the context of other patients' viruses may be more useful. For example, an IC_50_ change of 15-fold would be within the expected dynamic range for most NNRTIs, but would be considered exceptionally resistant if it applied to tenofovir. Further, since a majority of vPhenotypes collected fell below the lower Virco clinical cut-off defined for each drug, it may be additionally helpful to examine the distribution of vPhenotypes within a population in order to better resolve differences in resistance amongst patients. This approach may be especially useful in the case of newly released drugs, where clinical outcome data associated with the vPhenotypes are unknown.

A drug's vPhenotypic dynamic range provides a useful framework for interpreting vPhenotypic resistance data and captures information about the population distribution for resistance, which may also be useful. Note however, that its relevance for patient outcomes is not known and that the dynamic range of a drug may not necessarily reflect its clinical utility. 3TC/FTC susceptibility is dramatically decreased by a single mutation (the M184V mutation in reverse transcriptase [19]), giving a large dynamic range. Nevertheless, these are very effective, commonly prescribed agents. Similarly, there is no direct link between dynamic range and clinical outcomes for the PI drug class, since boosted-PI therapy is associated with better outcomes than treatment with non-boosted PIs, even though the patient's virus exhibits the same vPhenotype value for both.

The prevalence of resistance to specific drugs among patients failing therapy in our cohort largely reflects local prescription patterns over the past decade, as well as the introduction of new drugs over the years [20]. This also had an effect on the number of samples exposed to different drugs, with many samples (N = 18,392 of the total 19,611) exposed to the first antiretroviral drug, AZT, but fewer for recently approved drugs such as ETR (N = 1,545). Also playing a role are single-mutations that confer drug resistance, such as is seen for 3TC/FTC and the M184V mutation [20]. Drug resistance patterns as a whole also changed over time, with fewer cases of resistance in more recent years [21]. In addition, the specific Virco vPhenotype version has also changed over time, which may have influenced our results (though in general, the ranges were similar for all two year periods from 2000 to 2008, with the main exceptions of lopinavir and atazanavir, reflecting the introduction of widespread use of these agents; Figures S1, S2, S3).

The maximum changes in IC_50_ observed here were generally lower than those reported for “real” phenotypes determined using a recombinant viral assay (Virco Antivirogram® [22]). This is mainly due to the fact that a virtual phenotype is an average of fold-changes in IC_50_ seen in database samples with similar mutational profiles, while the actual phenotype assay will give a physical result of the ability of the virus to grow in the presence of a drug.

These data have enabled us to generate the range of phenotypes for each antiretroviral agent into which the vast majority of HIV patients receiving treatment in British Columbia will fall. As such, we believe this approach provides an objective framework for interpreting drug resistance that puts all drugs within a clinically relevant context and allows for the establishment of standardized guidelines for the application of drug resistance data in clinical practice. Although the data presented here represent a time-dependent phenomenon that reflects the standard of HIV care in British Columbia, these data could be regularly updated and this approach could provide a new avenue of analysis for international data.

## Supporting Information

Figure S1 NRTI dynamic ranges over time.
(TIF)

Figure S2 NNRTI dynamic ranges over time.
(TIF)

Figure S3 PI dynamic ranges over time.
(TIF)
