# Supplementary figures and images for: “Dynamic Range” of Inferred Phenotypic HIV Drug Resistance Values in Clinical Practice
Source: PLoS One. 2011 Feb 24;6(2):e17402. doi: 10.1371/journal.pone.0017402 (PMC3044728; doi:10.1371/journal.pone.0017402)

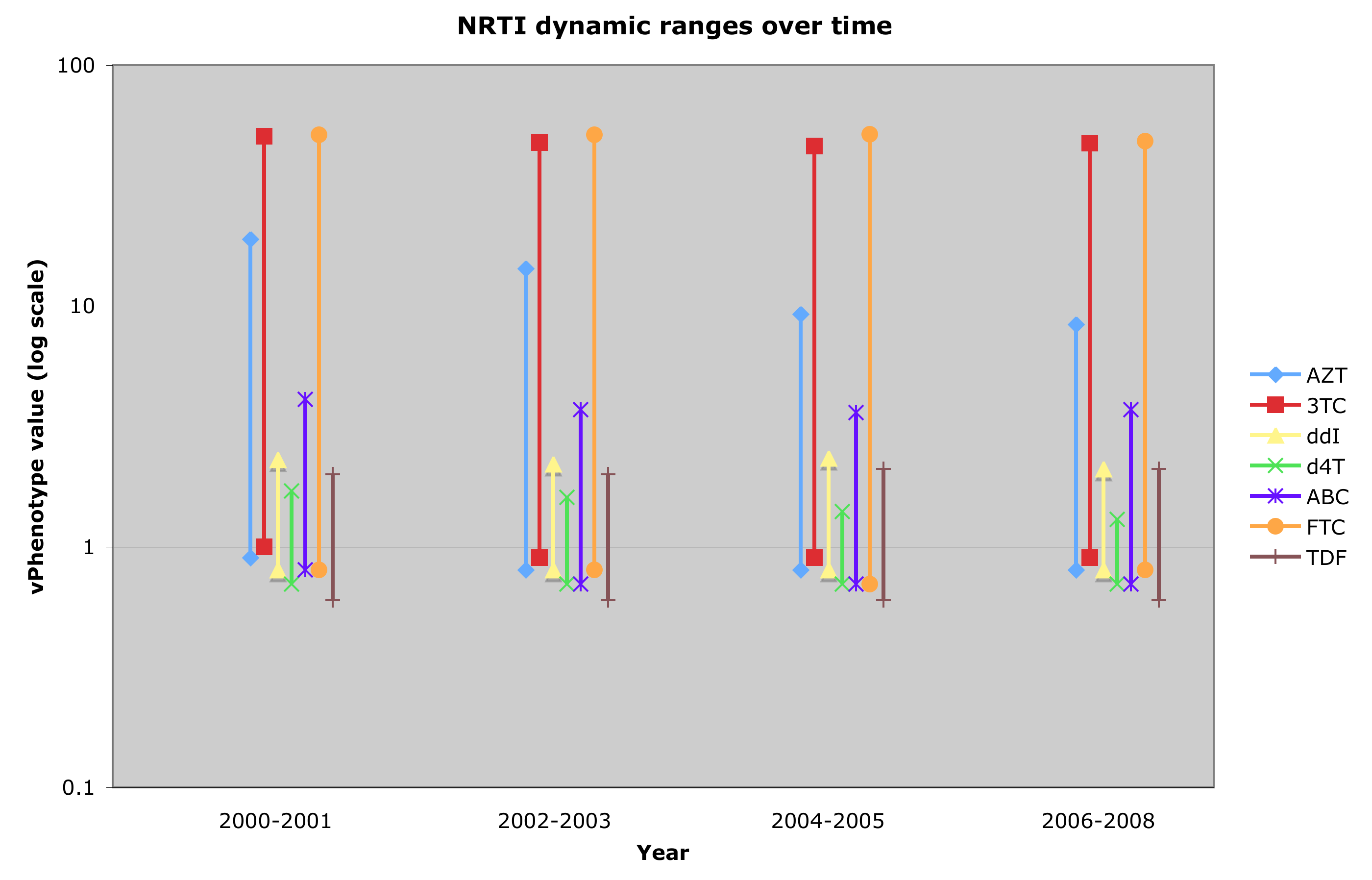

Supplement: Figure S1 — NRTI dynamic ranges over time. (TIF) [file pone.0017402.s001.tif]

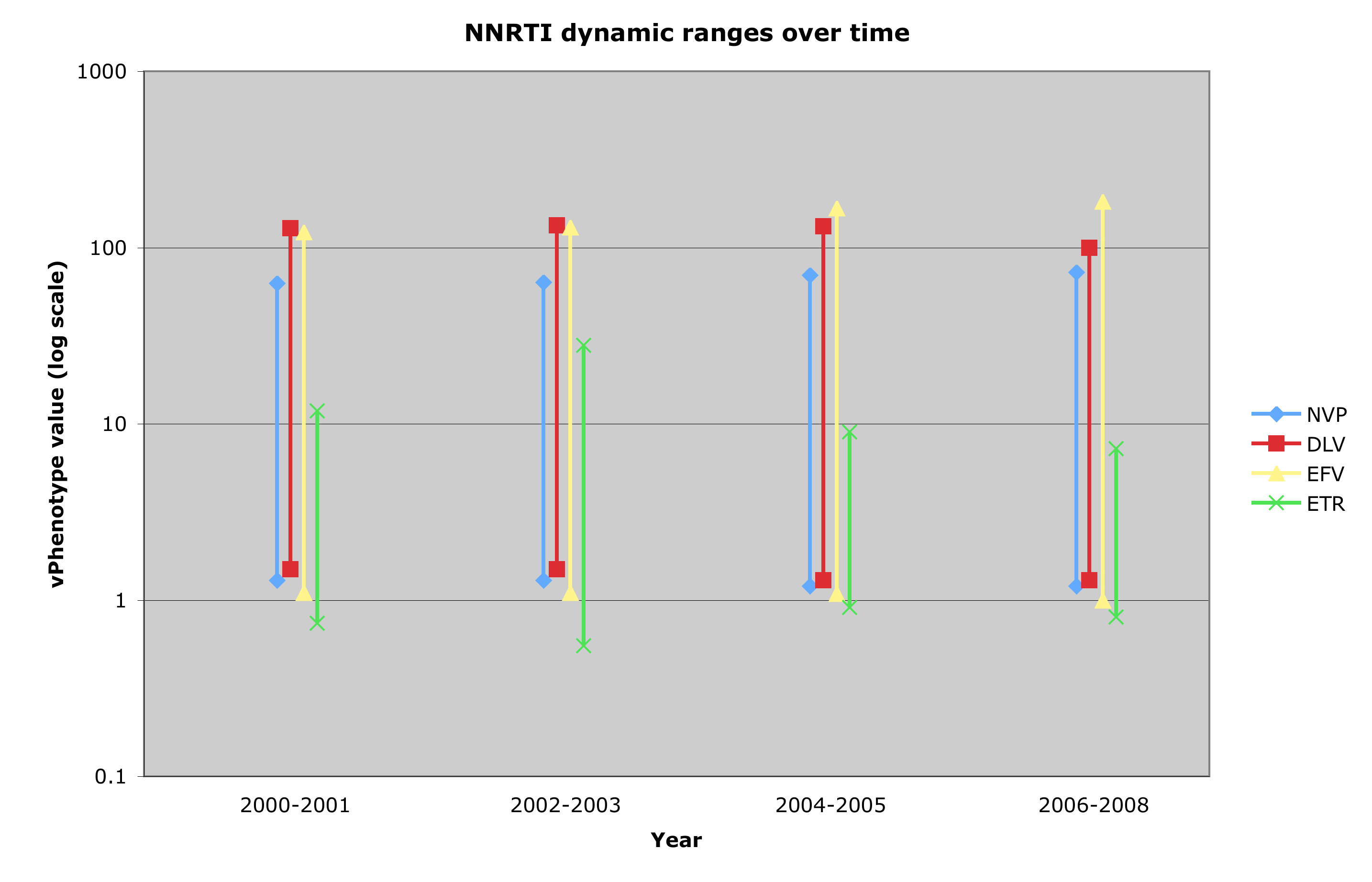

Supplement: Figure S2 — NNRTI dynamic ranges over time. (TIF) [file pone.0017402.s002.tif]

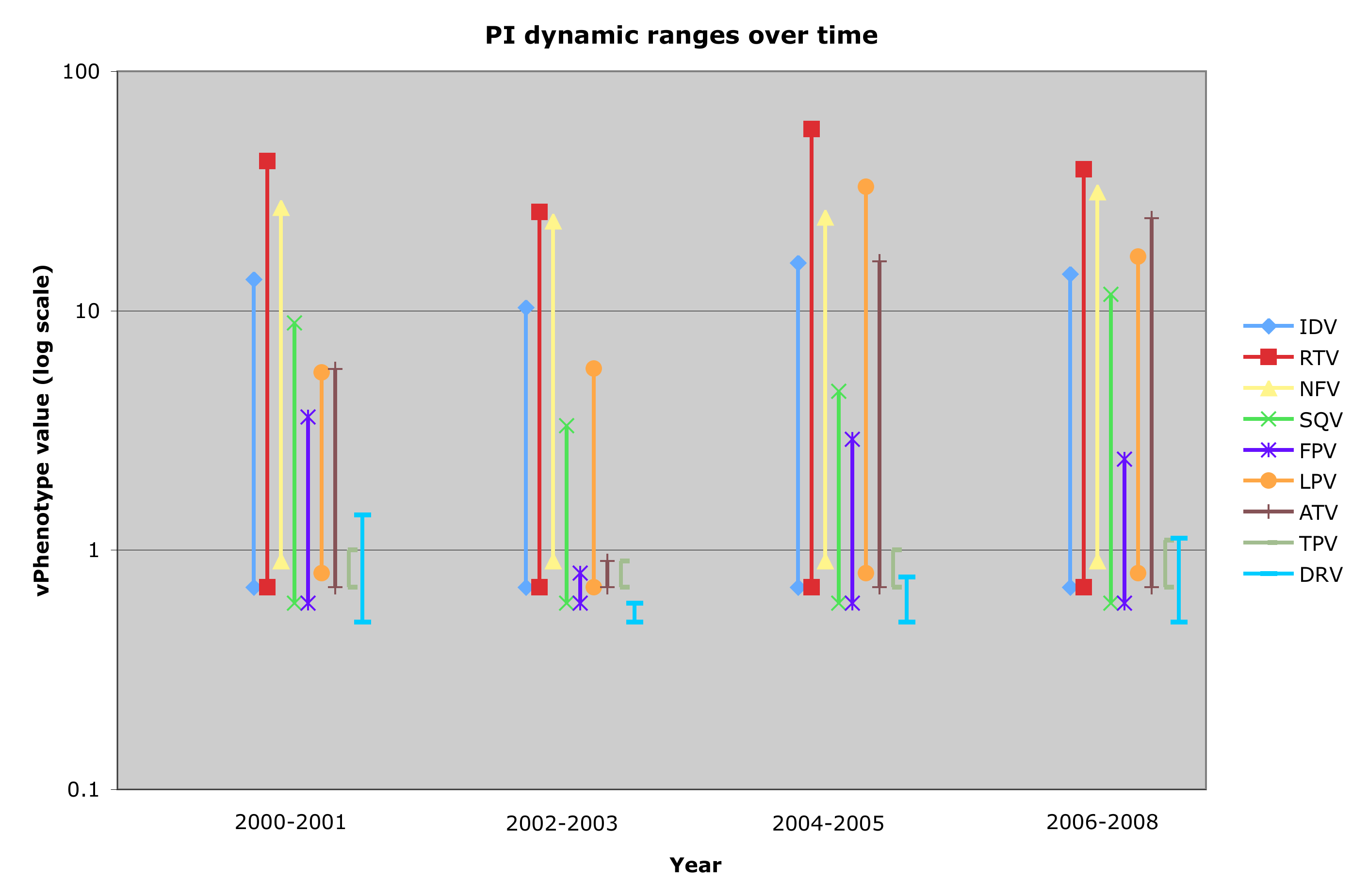

Supplement: Figure S3 — PI dynamic ranges over time. (TIF) [file pone.0017402.s003.tif]
